# Some Studies on the Anatomy of the Teeth and Their Practical Application1Read before the Odontological Society of Great Britain, May, 1905.

**Published:** 1905-09

**Authors:** 


### Reviews of Dental Literature.

SOME STUDIES ON THE ANATOMY OF THE TEETH AND THEIR PRACTICAL APPLICATION.[1] By Dr. W. D. Miller (Berlin).

MR. PRESIDENT AND GENTLEMEN OF THE ODONTOLOGICAL SOCIETY OF GREAT BRITAIN,—It gives me great pleasure to meet with you again, and I beg to thank you heartily for your cordial reception.

Under the risk of trespassing too often upon your valuable time, I have asked the permission to present a few slides for your consideration, relating to the hard new formations of the dental pulp. Every practitioner of dentistry is familiar with the fact that these formations often very seriously complicate the treatment of diseased conditions of the teeth. The studies I have the honor to present to you were undertaken under the supposition that more exact information regarding the frequency, situation, and character of these formations, apart from the scientific interest connected with it, would bring about a more thorough understanding of the difficulties encountered and of the manner of dealing with them.

[Dr. Miller proceeded to throw upon the screen, by means of the epidiascope, a series of very beautiful preparations and lantern slides from photomicrographs showing hard new formations of the pulp in varying positions and extents, a few of which he described in the following manner.]

The hard new formations of the dental pulp occur almost exclusively as secondary dentine and pulp-nodules (pulp-stones, internal odontomes, odontheles, denticles, etc.). The more or less diffuse calcifications frequently found in the pulps of senile teeth and in pulps which have suffered from some nutrient derangement are brought about by a simple precipitation of lime salts in the organic matrix of the pulp, and are not entitled to the name of new formations. Secondary dentine may be found in all cases where the cusps of the teeth have been worn down sufficiently to encroach upon the dentine, and usually in cases of chronic caries

---

[1] Read before the Odontological Society of Great Britain, May, 1905.

or other chronic irritations. In the first-named position it does not materially interfere with the process of cleansing the pulp-chamber or root-canals. In the case of chronic caries at the neck of the tooth, however, it may be found in such masses as to obliterate a considerable portion of the pulp-chamber. Where there has been much recession of the gums and a chronic irritation of the root, a formation of secondary dentine may be found at the beginning of the root-canal and very seriously impeding the entrance to the same. The next specimen illustrates a case which I, and no doubt many of you also, have occasionally met with in practice. The patient does not complain of acute pain, but rather of an occasional grumbling, so that he is repeatedly made aware of the fact that the tooth is there, and every time he visits his dentist he again calls his attention to it. The tooth will be found to give no reaction on application of heat or cold, and leaves us to conclude that there must be a dead pulp present. In attempting to treat a tooth of this character some time ago, I bored, as it seemed to me, at least half-way to the apex, and failing to come into contact with the pulp or root-canal, I desisted, and filled up the canal which I had bored with gutta-percha. In such cases we have to do with a more or less complete obliteration of the pulp-chamber and root-canal by a new formation of dentine. In the following specimen there is a still more extensive dentinification of the pulp; in fact, every trace of pulp-tissue to within about two millimetres of the apex of the root had been transformed into a dentine-like tissue.

Those hard new formations, designated as pulp-nodules, are found either embedded in the tissue of the pulp (free) or attached to the pulp-chamber or root-canal (parietal), or in the midst of the dentine itself (interstitial). Regarding the genesis of these pulp-nodules, you are no doubt familiar with the inversion hypothesis of Wedl. Wedl supposed that the layer of odontoblasts, through some cause or other, formed a fold, which dipped or projected into the pulp pretty much as the stratum Malpighii does into the embryonal tissue of the fetal jaw to form the enamel-organ. This fold may become detached from the membrana eboris, and is then in a position to form a free pulp-nodule, or it may remain in connection with it, in which case it forms a parietal nodule, which may subsequently become free by the absorption of the neck attaching it to the wall of the pulp-chamber.

This hypothesis is completely at variance with the experience of all who have made an extended study of pulp-nodules. While it is a most easy matter to find pulp-nodules (according to various estimates, from ten to thirty per cent. of all pulps containing them), no one has ever yet found, among the thousands of pulps that have been microscopically examined, such an inversion of the odontoblast layer as is described by Wedl and his followers. Nor have I, in the hundreds of pulp-nodules of varying sizes that I have examined, ever yet met with a trace of any process allied to resorption.

Personally, I have no doubt at all that all pulp-stones are found free and afterwards become attached to the wall only when it encroaches upon them or they upon it.

Pulp-stones may be limited to the crown portion of the pulp, or they may extend far into the radical portion, and I have occasionally found them quite at the apex. A condition is frequently found in senile teeth in which the gradual growth of a number of pulp-stones has led to their coalescence and union with the wall of the pulp-chamber. In all such cases we experience a special difficulty in devitalizing the pulp, as well as in its subsequent removal. The majority of pulp-stones show a lamellar formation.

I may remark, in passing, that it has been my experience that where severe neuralgic pains have arisen from the formation of pulp-nodules these have been sharply angular in shape. I do not know whether this experience could be borne out by that of other practitioners. We all agree, however, that in the majority of cases pulp-stones do not give rise to any disturbance whatever.

I wish to call your especial attention to the section on the screen, made from a tooth in which a large part of the crown had been destroyed by caries, and the softening of the dentine had quite reached the pulp. We see evidence of extensive suppuration, and note that the pulp, apart from forming a number of pulp-nodules, has thrown up a wall of calcific matter by which it has separated the diseased from the healthy portions; a very remarkable process which shows that the pulp of the human tooth has a considerable power of recuperation, and is a strong argument against the practice advocated by many of devitalizing not only every diseased pulp, but even the healthy pulp accidentally exposed in excavating a cavity. This self-protecting process is very common in the tusks of the elephant, and I have met with it now five times in human teeth; one case has also been recorded by Gysi; and it seems to me that, in view of this evident recuperative power on the part of the dental pulp, we ought to be able in many cases to restore those pulps which have shown evidence of but slight derangements to a healthy condition.

The next specimen is also one which merits our consideration. It shows a condition which, I believe, occurs oftener than is usually supposed. Besides a small free nodule and diffuse formation of secondary dentine along the left margin, we see a large nodule which has become attached to the wall at two points. In attempting to cleanse such a root-canal, we might by good luck succeed now and then in passing a fine broach through the narrow canal at the left of the nodule; more frequently, however, it would catch, and if the tooth had been previously treated by a colleague we would suspect him of having left the point of a broach in the canal. It is in such cases always better to take a more charitable view of the matter, and consider, first, whether we have not to do with a condition similar to that shown by the specimen.

The next slide illustrates a class of cases which, in my estimation, are among the most difficult with which we have to deal. We have here one of the roots of a first permanent molar of a child about eight years of age. The apex is still wide open, and although the tooth is so young a number of pulp-nodules have already formed. One of these nodules has become united to the wall of the root-canal, and every attempt to clean out the pulp-canal below this point would probably only result in pushing its contents farther toward the apex, where, in at least nine cases out of ten, it will sooner or later give rise to serious disturbance. The time may come when radiography will be so simplified and perfected that we may be able to detect such a condition of affairs, and, by boring away the nodule, succeed in removing the contents of the canals below that point. At present, however, we are rather working in the dark when we are treating conditions of this character. Under the most favorable circumstances teeth with open foramina are very difficult to treat successfully. We have here, in my estimation, the only case in which iodoform can be used to advantage.

The next slide shows a condition which is often present, and which may be the cause of our lack of success in treating certain cases. It is a longitudinal section through the root of a lower bicuspid, in which the root is much contracted longitudinally, and there is an adventitious pulp-canal on one side which is inaccessible to treatment, and may give rise to disturbance at any time.

[Dr. Miller further showed specimens of interstitial pulp-stones in human teeth, of pulp-stones in milk-teeth, and diffuse calcification of the pulp, in which he called especial attention to a preparation which had been presented to him by Mr. Mummery.]

The following method was recommended for bringing pulp-stones very clearly into view: Grind the teeth down from both sides sufficiently to expose the pulp, then harden them in formalin or alcohol, impregnate with Canada balsam, after the method of von Koch and Weil, and after the balsam has become completely hard grind them down to the desired thickness and subject them to the action of a one per cent. solution of hydrochloric acid for from one to two minutes. Then wash them and bring them into the staining solution, and we shall find that dentine and all the calcifications and pulp-stones, and so forth, which are formed in the pulp, will be stained bright red, or whatever color we have chosen, while the pulp-tissue remains unstained. You will see also that these pulp-stones can be brought to view very easily without the use of the microscope, and without any photography.

[In conclusion, a number of natural sections of the teeth and jaws of human beings and of animals, which were particularly interesting from the point of view of comparative dental anatomy, were thrown on the screen.] Sections from the teeth of the horse, warthog, waterhog, etc., can be easily obtained by first dividing the teeth into sections about three or four millimetres thick by means of a knife-edged corundum wheel about two and one-half inches in diameter, and then grinding the sections down to the desired thickness on a corundum wheel about ten to twelve inches in diameter, mounted horizontally and driven by a motor of one-eighth to one-fourth horse-power. For holding the sections while grinding, a piece of hard cork is far preferable to any means which have hitherto been devised. After grinding, the sections were brought for from one to two minutes into a two per cent. solution of hydrochloric acid, whereupon they were washed with water and stained in eosin, picrocarmine, or triacid. Very good results may also be obtained by staining in a thin solution of thionin for twenty-four hours, then subjecting to a concentrated solution of picric acid diluted with equal parts of water for about half a minute. Striking results may often be obtained by a simple staining with eosin. The enamel remains white, the cement becomes red, while the dentine takes on a bluish tinge.

[Dr. Miller referred to an attempt which he had made in conjunction with, and following out a suggestion of, Mr. Mummery, to find a quick and simple method of preserving the pulp in ground sections.] It is a matter of great difficulty to grind sections of teeth and preserve the hard and soft tissue at the same time. To do that we are obliged to impregnate the tooth with Canada balsam and harden it, which takes six or eight weeks. Mr. Mummery suggested the possibility of freezing the teeth and grinding them, and we carried out experiments which were fairly successful. We placed the teeth in a drop of water on the upper side of a metallic box, froze them by means of chloride of ethyl, and kept chloride of ethyl playing upon it while grinding. You see from the sections on the screen that the results are fairly good, but as we were at an expense of sixteen shillings for chloride of ethyl in making four sections, we left off our experiments for the time being. We hope, however, to take them up again later.

I thank you, Gentlemen, for your kind attention.—*Transactions Odontological Society of Great Britain.*

